# In this month’s Bulletin

**DOI:** 10.2471/BLT.21.000821

**Published:** 2021-08-01

In the editorial section, Oladayo A Afolabi et al. (542) point to requirements for increased palliative care capacity in the African Region. Lena Morgon Banks et al. (543) describe the insurance coverage needed to improve access to health care for people with disabilities. 

In the news section, Gary Humphreys (546–547) reports on the development of COVID-19 passes and the technical, ethical and social issues affecting their use. Amal Saif Al-Maani talks to Andréia Azevedo Soares (548–549) about antimicrobial resistance and the need for multisectoral approaches to mitigating its development.

## Canada, Democratic People's Republic of Korea, Ethiopia, Mauritius, Pakistan, Philippines, South Africa, Sudan, Turkmenistan, United Kingdom of Great Britain and Northern Ireland, United States of America

### Water, sanitation, hygiene as part of antimicrobial resistance strategies

Sabiha Essack (606–608) notes the countries that cover both sectors in national action plans.­

## France, Germany, Italy, Japan, Spain, Switzerland, United Kingdom of Great Britain and Northern Ireland, United States of America 

### How many antibiotics?

Bryony Simmons et al. (550–561) measure progress towards antibiotic-use targets.

## India

### Watching for antimicrobial resistant pathogens 

Sonam Vijay et al. (562–571) describe an integrated surveillance network.

## Global

### Providing mental health interventions 

Suzanne M Connolly et al. (572–582) review the evidence for lay counsellors.

### Arboviral infections and waterborne diarrhoea

Hans J Overgaard et al. (583–592) make the case for integrated disease management.

### Improving population-level physical activity 

Tracy Nau et al. (593–602) propose a framework for legal strategies. 

### Pandemic prevention

Harman S Sandhu et al. (603–605) consider the impact of animal husbandry on emerging pathogens.

